# Cowpox Virus Transmission from Pet Rats to Humans, France 

**DOI:** 10.3201/eid1505.090235

**Published:** 2009-05

**Authors:** Laetitia Ninove, Yves Domart, Christine Vervel, Chrystel Voinot, Nicolas Salez, Didier Raoult, Hermann Meyer, Isabelle Capek, Christine Zandotti, Remi N. Charrel

**Affiliations:** Université de la Méditerranée, Marseille, France (L. Ninove, N. Salez, D. Raoult, R.N. Charrel); Assistance Publique–Hôpitaux de Marseille Timone, Marseille (L. Ninove, D. Raoult, C. Zandotti, R.N. Charrel); Centre Hospitalier, Compiègne, France (Y. Domart, C. Vervel, C. Voinot); Centre National de la Recherche Scientifique, Marseille (D. Raoult); Institut für Mikrobiologie der Bundeswehr, Munich, Germany (H. Meyer); Institut de Veille Sanitaire, Saint Maurice, France (I. Capek)

**Keywords:** Zoonoses, viruses, cowpox, outbreak, pet rats, orthopoxvirus, humans, France, expedited, dispatch

## Abstract

In early 2009, four human cases of cowpox virus cutaneous infection in northern France, resulting from direct contact with infected pet rats (*Rattus* *norvegicus*), were studied. Pet rats, originating from the same pet store, were shown to be infected by a unique virus strain. Infection was then transmitted to humans who purchased or had contact with pet rats.

The recent trend of adopting wild animals as pets will inevitably create conditions favorable for emerging pathogens. Consequently, under the influence of increasing commercial enterprise, potentially highly pathogenic agents are likely to emerge and fuel unprecedented epidemic situations. Cowpox virus (CPXV) is a member of the family *Poxviridae*, genus *Orthopoxvirus*. In contrast to smallpox virus (exclusively human), the reservoir for CPXV, and possibly monkeypox virus, is believed to be rodents (*1**,**2*). CPXV is distributed in Europe, Russia, the western states of the former United Soviet Socialist Republic, and adjacent areas of northern and central Asia (*3*). Natural reservoir hosts of CPXV are wild rodents, such as bank voles and wood mice (*4**,**5*). Transmission to humans is through contact with infected animals, mostly domestic cats, which are occasional predators of wild rodents (*4**–**6*). We studied an outbreak of cowpox virus cutaneous infection among 4 human case-patients.

## The Cases

**Figure 1:**
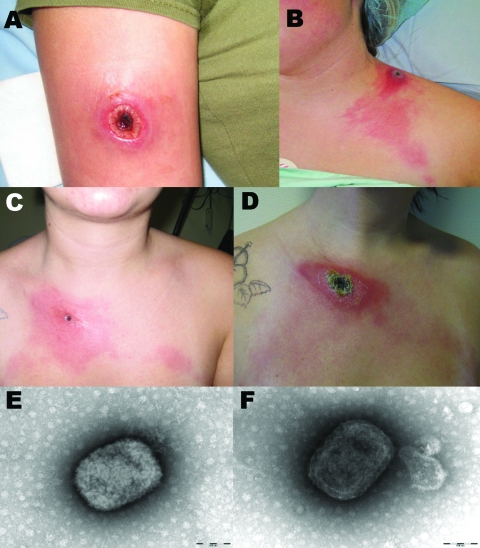
Cowpox virus infection in 3 persons in northern France caused by transmission from infected pet rats. Cutaneous lesions caused by cowpox virus are shown in patient 1 (A), patient 3 (B) and patient 4 (C, D). The 2 latter patients had lymphangitis associated with the local lesion. Panel C was obtained on January 30, 2009, panel D on February 6, 2009. Negative-staining electron microscopy showed mulberry forms with conspicuous but short, randomly arranged surface tubules (E) and capsule forms with deeper stain penetration (F), both highly suggestive of poxvirus. Scale bar for panels E and F = 100 nm.

Case-patient 1 was an 18-year-old woman. She was scratched on the right arm by a pet rat while visiting a friend who had several domestic rats (*Rattus norvegicus*). One rat had been purchased at the end of December 2008 from a pet store. The rat became sick with sneezing, conjunctival hemorrhages, and epistaxis; it died 4 days after purchase. On January 4, 2009, the patient sought treatment at the emergency department of Compiègne Hospital. The lesion was excised and the patient was treated with amoxicillin-clavulanate. However, the wound did not heal. On January 11, ofloxacin was added to the treatment regimen. Eight days later, the patient was admitted to the hospital with a black necrotic scab on the internal surface of the right arm, regional lymphangitis, and axillar lymphadenopathies (Figure 1, panel A). After 3 weeks of unsuccessful antimicrobial drug treatment, she underwent surgery to remove the affected area. The outcome was favorable.

Case-patient 2 was a 17-year-old woman who had purchased a domestic rat at the end of December 2008. The rat died within 3 days of purchase and had respiratory symptoms identical to those of the rat that scratched case-patient 1. Six days after the rat died, an inflammatory cutaneous macular lesion appeared at the base of the patient’s neck, causing local pain and intense inflammatory reaction. The patient was admitted to the emergency department of Compiègne Hospital. Amoxicillin-clavulate was prescribed, but the necrotic scab continued to grow, and local pain increased along with fever (39°C) (Figure 1, panel B). Surgery was performed and the outcome was favorable.

Case-patient 3 was a 14-year-old girl. She was admitted to the emergency department of Compiègne Hospital on January 14, 2009. On January 3, she had purchased a rat from the same pet store as case-patient 1. Soon afterward, the rat began to cough and show signs of hemorrhagic lachrymal oozing**;** the rodent died on January 6. On January 13, the patient had maculopapular lesions on the upper right eyelid, on her left shoulder, and at the base of her neck. Due to her deteriorating condition, she was admitted to the emergency department on January 17 with rash characterized by erythema and edema, and painful regional lymphangitis and lymphadenopathy. Surgery was performed and the outcome was favorable.

Case-patient 4 was a 29-year-old woman who reported having been scratched by a rat on January 21, 2009. An inflammatory macule on her clavicle had progressed through papular, vesicular, and pustular stages; she also had fever and malaise. On January 14, she had purchased a domestic rat in the same store as the 3 previous case-patients. The rat had respiratory symptoms similar to the previously infected rats and died on January 21. The patient was admitted to Compiègne Hospital on January 30; examination showed a 20-mm black eschar with a crust, regional lymphangitis, and painful lymphadenopathies (Figure 1, panels C and D). Outcome was spontaneously favorable.

Biopsy specimens of case-patients 1–3 were sent to the National Reference Center for Rickettsial Diseases (Marseille) on January 21 because anthrax and/or rickettsial disease was suspected. Broad range PCRs were performed for bacteria (16S rRNA) (*7*) and fungi (18S rRNA) (*8*). In the absence of etiology, and based on information provided by the Institut de Veille Sanitaire, negative-stain electron microscopy was performed on January 26. The biopsy samples showed typical poxvirus-like particles (Figure 1, panels E and F). Molecular diagnosis was performed by using PCR targeting a 260-bp fragment in the cowpox hemagglutinin gene (forward primer 5′-TACTTTTGTTACTAATATCATTAG-3′, reverse primer 5′-AGCAGTCAATGATTTAATTGT-3′). Direct sequencing of the PCR product identified cowpox virus by BLAST analysis (http://blast.ncbi.nlm.nih.gov/Blast.cgi) against the GenBank database. The virus was isolated by using monolayers of Vero cells in 12.5-cm flasks (*9*). When cytopathic effect was obvious, DNA was extracted from the supernatant, and the complete hemagglutinin gene was sequenced from a PCR product (forward primer 5′-CCATTGGAAAAAACACAGTAC-3′, reverse primer 5′-CCAAATATATTCCCATAGTC-3′), amplifying a 1,183-bp region. PCR protocols are available from R.N.C. Electron microscopy and PCR were performed on formalin-fixed scabs from the lesion of case-patient 4. Both test results were positive morphologically for a poxvirus and by PCR for cowpox virus.

**Figure 2:**
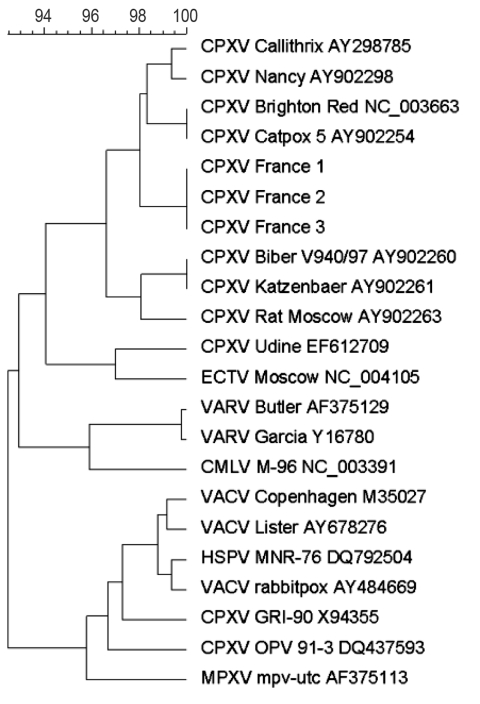
Phylogenetic tree based on nucleotide sequences in the hemagglutinin gene. Sequence information corresponds to virus acronym/strain/GenBank accession number. Phylogenetic study was conducted using MEGA software version 4.0 (www.megasoftware.net). Genetic distances were calculated with the pairwise distance method. Phylogenetic tree were constructed with the neighbor-joining method. CPXV, cowpox virus; ECTV, ectromelia virus; VARV, variola virus; CMLV, camelpox virus; VACV, vaccinia virus; HSPV, horsepox virus; MPXV, monkeypox virus. Scale bar indicates genetic diversity at the nucleotide level.

The virus was isolated from serum samples of case-patients 1–3 and assessed by cytopathic effect on Vero cells, electron microscopic morphologic identification, positive PCR amplification, and subsequent direct sequencing. The 1,183-bp PCR showed positive results for each of the 4 specimens. Sequences were deposited in GenBank under accession nos. FJ754355–FJ354357. A 1,047-bp sequence was definitive and used for fine comparative genetic analysis with the full-length hemagglutinin gene sequences available in GenBank. The most closely related sequence corresponded to clone cow HA24 of the catpox 5 isolate of cowpox virus, isolated from a cheetah in 1982 in the United Kingdom (AY902254). Genetic heterogeneity with Y902254 comprised 15 mutations; 10 were nonsynonymous, and 3 were insertions. Phylogenetic analyses indicated that sequences corresponding to these cases grouped together and were clearly distinct from other cowpox virus strains previously reported (Figure 2). Sequence analysis indicated that these 4 infections were caused by a virus strain distinct from other cowpox virus sequences retrieved from GenBank.

## Conclusions

Sporadic human cases of cowpox virus infection have occurred in several European countries over the past few years. For most cases the source was domestic cats (*4**–**6**,**10**,**11*). Rat-to-human transmission of cowpox virus was described in the Netherlands, but the source was a wild rat, not a pet rat (*12*). We know of only 1 previous case of human cowpox virus infection that may be linked to a pet rat (*13*). In the 4 cases reported here, the rodent host was clinically sick and rapidly died. All 4 patients reported scratches caused by rat claws, not bites, while handling the rats as pets. In 3 of the 4 cases, fever (39°C) was noticed at the pustular stage, associated with lymphangitis and regional adenopathies. Interviews with the 4 case-patients showed all had purchased or had been in contact with domestic rats originating from the same pet store. Further investigations traced the origin of the cowpox virus-infected rats to a rat breeder in the Czech Republic (*14*).

Recently, similar human cases linked to contact with pet rats have been reported in France, suggesting that the outbreak may involve more cases than were initially realized (*14*). The situations in France and Germany mimic the monkeypox outbreak in the United States, i.e., human transmission of the virus by pet prairie dogs contaminated by probable contact with Gambian rats imported from Africa and directly associated with a US pet retailer (*15*). Our study and the US outbreak emphasize the need for extreme caution when humans adopt animals of exotic origin as pets. Our study justifies the establishment of a national diagnostic capability and the corresponding human expertise to enable rapid diagnosis and identification of human pathogens that can cause unimaginable levels of disease in our communities.
